# East and West

**Published:** 1887-11-19

**Authors:** Leslie Keith

**Affiliations:** Author of "The Chilcotes," "Uncle Bob's Niece," etc.


### EAST AND WEST.

By Leslie Keith.

Author of "The Chilcotes," "Uncle Bob's Niece," etc.

(*Continued from p.* 120.)

Chapter VIII.—An Old Friend with a New Face.

"I came," said Mervyn, obeying her invitation, and concealing his surprise successfully, "to ask Miss Jardine to come to my rescue, as she has done before. There's a fellow downstairs with a broken head, and a wife who can do nothing more sensible than cry over him."

"I will go with you," said Alicia promptly. "Not Mary; she is tired."

Mary had remained standing, but she uttered no protest. When Alicia spoke in tones of command she was instinctively obeyed. The young man looked at her with a veiled smile that was more or less incredulous, but he did as he was bid—that is, he held the door open while she walked out of the room. She turned round on the narrow stair and faced him.

"Is it the man from the country?" she asked.

"It is the man from the country—otherwise William Ford. He has got into a—in short, into trouble. All I want is, that his wife and children shall be taken out of the way while I patch him up."

"Very well," said Alicia with conciseness, following him into the room. The nature of Ford's "trouble" was apparent even to her ignorance—only she would have called it by a severer name. He lay on the floor, breathing heavily, and bleeding from a wound in the head; while his wife—a young woman whose youthful prettiness had faded under early-coming cares—hung wailing over him; her children clinging to her skirts, and making the lamentation into a chorus with their frightened cries.

Alicia got them away, she scarce knew how. She enticed the babies with her rings and trinkets, and she awed the feeble mother into silence by the unexpectedness of her appearance. So fine a lady had never before been seen in Baron Street.

As for Alicia, while she talked she was filled with a great wonder. How came Felix Mervyn to be there? She had met him last in very different scenes. How long ago? A year? Perhaps two, or three, or even four. One year in Mayfair is very like another, and people come and go, and are quickly forgotten. They had known each other well in those days, and had been attracted by a mutually-shared cynicism that turned the world into a bitter jest for their sport. From all this on a day he had passed—vanishing, as it seemed, without a farewell; she had seen him no more, and had scarcely thought of him, indeed, till she found him now binding up the head of a drunken man, and doing it with the skill and cool confidence of a practised hand.

If he had any similar train of thought as regarded her, he gave no sign of it by so much as a glance in her direction. He did what he had to do, and when he looked up it was to address the scared wife.

"He's all right," he said; "no need to cry over him yet. You let him rest a bit, and I'll look in this evening, and see how he's getting on."

"Oh, doctor," she said falteringly, "there isn't no evil in him—he were allus good to me an' the weans. He were fair clemmed, an' the drop o' drink a neighbour gien him ——"

Alicia listened thus far in silence, and then she walked out of the room. When Mervyn followed her at his leisure he found her standing on the landing. He looked at her half smilingly.

"You disapprove of my patient?" he said; and in answer to her gesture he went on lightly: "he is a most reprehensible person, no doubt; he had no breakfast this morning, and no dinner yesterday, nor the day before either, very likely; and yet when a neighbour gives him a drop of beer he hasn't the strength of mind to refuse it."

"He had work," she said hardly.

"He earned the luxurious wage of 24s. a week, 6s. of which went for rent. That left the handsome income of 18s. to feed, clothe, and warm four people, yet he hasn't laid by a fortune, and now that he is out of work —— "

She put up her hand to stop him.

"The woman and the children are in want of food," she said. "Oh, no, I am not pitying them, but I dislike to hear children cry; it is unpleasant to me. If they had bread and milk they might stop crying. Where can I buy these?"

"Those luxuries are to be had even here—by the happy few who can pay for them." He led the way downstairs, and she followed him, walking with her head erect.

The loungers were still in the streets; the men, propped against the walls, were smoking pipes— that being apparently the sole business of their lives; the women engaged in loud-voiced talk from open doors and windows. These women, she noticed, were slatternly, and looked as if they never washed or used a brush and comb. The fashion of their garments was archaic, and suggested a strange absence of feminine vanity; their voices were coarse, and the looks turned on her fell short of being respectful. Such remarks about herself as she passingly caught were full of envy, hatred, and malice. If she had been one of them selves they would have stood by her loyally, and helped her with ungrudging generosity; but she was not one of them; she was of the order whom it comes natural to them to hate. She observed, however, that Mervyn's escort protected her from open insult. He was tolerated; his nods and lively sallies were even surlily returned. He had something to give, and in the East-end that is the standard by which most intruders from other regions are judged. The young lady might have something to give too; but she looked proud, and pride is an unpardonable sin here, where all men—and women—are equals.

She borrowed a jug and tumbler at the dairy, paying for their use with a careless indifference that bespoke a full purse. At the baker's she laid in a liberal store, and then she demanded to be taken to a grocer's.

Mervyn remarked that there was not one quite at hand, and this brought a question to Alicia's lips.

"There are very few shops—no butchers, or fishmongers, or greengrocers. How is it?"

"They don't particularly want to starve, I suppose," Mervyn laughed, "and they would find no custom here. We none of us have any money, and when we can scrape a few pence together it is the coster we patronise. Twopence spent on bones is considered a handsome outlay, and it is economical too, for when you've had your soup or your stew you can resell the bones for a halfpenny. I learned that useful fact from an old lady whose housekeeping expenses amounted to six farthings for every week-day, and seven for Sundays."

Alicia digested this information in silence; perhaps she was telling herself it was not true. Mervyn carried her parcels and walked at her side, wearing a fine air of gravity.

"How was the Duchess when you saw her last?" he asked politely. "Lady Mary is doubtless married; and Miss Kitty—who is her latest victim? I find myself four years behind the world. If I use the wrong phrases and resuscitate buried stories, you must forgive me."

Alicia looked at him with a side long glance.

"They called you 'doctor,'" she remarked abruptly.

"I am legally permitted to claim the distinction," he said modestly.

"Do you come here often?"

"I came here four years ago; I am here still. If you will pardon my saying it, I find the new world more interesting than the old. It amuses me—it interests me. The old women are more entertaining, and even more frank, than the dear Duchess. The men do not exhibit the restlessness that was our bane in the other world; they don't care to be clean, or honest, or thrifty, or industrious; they don't even care to be amused. Think how immensely that simplifies life. I am not bored here at all; I am supported by a constant sense of humour. I like it; it entertains me; that is why I am here."

If Miss Frankland recognised in these phrases a subtle caricature of her own habitual refrain, she found no answer to it. She took her parcels from him at the foot of the stair, and, lingering there a moment, she heard him go quickly down the street. His step had cheer in it, and the voice was gay in which he chaffed the women and rallied the men. And this after four years—four years of Baron Street!

When she had fed the feeble young woman and her children —who were almost too dazed to be properly grateful—she carried what was left of the bread and milk upstairs. Mary did not refuse to share it with her, and while they ate, Alicia had more questions to ask; but this time they concerned Felix Mervyn.

"Do you see him often?" she asked.

"He comes to see the young man upstairs—it was there that I first saw him. He is here and there, wherever people need him, and always laughing and bright, putting some heart into the poor, sad people. That is what a doctor should be—down here."

"He was not very merry when I knew him, and yet he had everything to make him so. He is the son of a baronet —to be sure he is a younger son, but he has means: money enough to live as he likes and go where he likes. And yet he cuts himself off from everything—society, clubs, civilisation in short, and buries himself here."

Mary looked up with a light in her gentle eyes.

"If there were more like him," she said, "more noble men who could forget everything but our common brotherhood, and who would come down to this unknown world and cast in their lot with it, there would not be the sin and the wickedness and the hardships and the cruelties that make life one long bitterness to the poor. Oh, I know something of it now," said Mary, pausing a moment, and clasping her hands. "Down in the country there was no one more careless, more selfish than I. It broke my heart to leave the green fields and the silent hills. I hated the streets. I hate them still." She hung her head. "But when I think of the people here, and of my own happier lot, and of all that was given me these many years, I am ashamed—ashamed."

Alicia started up and began to pace the hot and airless little room fiercely.

"Mary," she said, at last finding words, "come home with me. Come and see our London before you judge us."

"I do not judge you."

"You would have no right," said Alicia with pride. "You know no more of the life that I have lived than you knew of this before you came here. Your hills could not help you to imagine it, any more than they could picture this to you. You cannot do us justice unless you come and live awhile with us, and see for yourself."

This sounded a little like the stirring of a guilty conscience. Who had been accusing this proud young lady, unless it was herself? Had Mary's very presence—set against this joyless and melancholy background—a tacit reproach, as Kitty indeed had found it?

"I am glad it has been—I am glad it is—different from this," said Mary gravely. "That it is beautiful and easy needn't hinder it from being good and useful; it is the wretchedness and hopelessness here that make people bad."

Alicia laughed a bitter little laugh.

"If you had pretended to know anything of our ways," she said, "that little sermon of yours would have betrayed your ignorance. Goodness and usefulness do not concern us at all; they are not amusing, and our one passion is to be amused. They are tedious; they interfere with pleasure! Goodness, of the kind you mean, doesn't flourish in Mayfair. If you imagine we are given over to charitable works——"

"Some of you are good, I think," said Mary with conviction. "Some of you are charitable, else you would not be here. You would not have comforted that poor woman, and fed her and her children. You would not have sought me out."

"Hush!" said Alicia, almost sternly. "I have told you before that I am not moved by any high motive. I am not the least interested in these people; on the whole, they disgust me. If I fed the children, it was only to make them quiet: their wailing was infinitely disagreeable to me, and I took the quickest way to stop the annoyance."

"That doesn't account for your coming to see me, and helping me to sew," said Mary with a little smile.

"You are a relative," said Alicia loftily, as if the honour of alliance with the Frankland family sufficiently covered all Mary's drawbacks. "And perhaps, if you had not been what you are, I should not have come again. I have no wish to pose as a philanthropist. I came here as a student of human nature." The phrase sounded so pompous to her ear that she was bound to laugh. Mary laughed too, and this little burst of gaiety somehow wholesomely cleared the air.

When Alicia next spoke her tones were almost pleading.

"Mary, come home with me," she said. "I am all alone; I have the house—and indeed Park Lane—to myself; nobody will come near us, since there is nobody to come. You shall do as you like—you shall even sew aprons if it pleases you. It will be quieter than it is here, and fresher, and possibly"—she looked round her—"a trifle less ugly." Then her tone became a little more urgent. "You ought not to be here," she said. "It may be right for others—for men or for Bible-women and people like that; but it is not right for you to live here. You can't do any good; it is a needless and a useless sacrifice. Come home with me."

"No," said Mary, shrinking back, but speaking resolutely. "I couldn't live your life. I am not a lady. I am poor as these people; I am one of them; we have the same struggle and the same difficulties. If I went away into your world, and tried to live, as you say, in quietness and peace, with beautiful things about me, their faces and voices would haunt me; it is an anguish to be here—to see and hear, and to be able to do so little; but it would be a worse pain if, having known this, I were to choose ease and forsake my neighbours in their trouble. And besides"—her voice took on a new, vibrating tenderness—"there is David. You have not seen him or you would not have asked me to go with you. I can never leave David."

At the mention of his name Alicia's purpose turned cold within her. She could very well have spared this unknown cousin, and she had no impulse to include him in her invitation. Mary had a natural grace and refinement that would befriend her in any position; but so much could not be hoped for from a country farmer. Alicia knew very well that an inborn instinct often helps a woman to rise above her surroundings and to practise the arts of civilisation, while her father and brothers are still content to be barbarians. Mary, in spite of her modest disclaimer, was a lady, but it was not in the least likely that David was a gentleman. She thought of the rough young countryman, of manners "barren and untilled" as his own hills—with a taste in jokes, no doubt, that she could not share—perhaps with even more impossible views on the subject of their common cousinhood; and as she conjured up this mental vision she shut and barred the doors of Park Lane against him. Something of the old pride and scorn returned to her.

"As you will," she said indifferently. "If you prefer this——"

"I don't prefer it. If I did, it would not be so hard. Please don't think me ungrateful," said Mary penitently. "I thank you more than I can ever tell you for coming of your own will to see me—for giving up a whole day to me."

"Oh, as to that, I mean, if you will allow me, to come to see you every day—since you will not visit me. If it were not that I promised papa to sleep at home every night, I should have asked you to get a lodging for me—it would have saved some trouble. But I have given my word, and must keep it."

"You couldn't have lived here; your father was quite right."

"You have lived here for months, and I don't suppose anything very dreadful has happened to you."

"But I am different," said Mary, looking distressed—"quite different; and I think you had better not come here again."

"Why not?" asked Alicia haughtily.

"Because I don't think you realize to what it may lead. If you come here every day, as you say you will, there may come a day when you will not find it possible to forsake us. That is the price one pays. One cannot come and go again, and forget it all."

"Oh, I'm not afraid," said Alicia calmly. "You forget that I come simply a student, an observer of the race. When I have finished my studies, when I cease to find them amusing, I shall go away again, and forget you without difficulty."

This was the view Felix Mervyn had taken of Miss Frankland's unlooked-for advent. She had come to amuse herself with the barbarians of the East, but her fastidiousness would not allow her to be long amused. The new pastime was not likely to outlast the day, and he had bidden her good-bye with no expectation of seeing her again.

Whether had he or Mary read her the more correctly?

(*To be continued.*)

